# Exploring the Effects and Potential Mechanisms of Hesperidin for the Treatment of CPT-11-Induced Diarrhea: Network Pharmacology, Molecular Docking, and Experimental Validation

**DOI:** 10.3390/ijms25179309

**Published:** 2024-08-28

**Authors:** Xinyao Shu, Ruitong Xu, Peiyu Xiong, Junyu Liu, Zubing Zhou, Tao Shen, Xiaobo Zhang

**Affiliations:** School of Basic Medicine, Chengdu University of Traditional Chinese Medicine, Chengdu 611137, China; shuxinyao0341@163.com (X.S.); xuruitong@stu.cdutcm.edu.cn (R.X.); xiongpeiyu@stu.cdutcm.edu.cn (P.X.); junyuliu1007@stu.cdutcm.edu.cn (J.L.); zhouzubing@stu.cdutcm.edu.cn (Z.Z.)

**Keywords:** chemotherapy-induced diarrhea, hesperidin, network pharmacology, molecular docking, experimental validation

## Abstract

Chemotherapy-induced diarrhea (CID) is a potentially serious side effect that often occurs during anticancer therapy and is caused by the toxic effects of chemotherapeutic drugs on the gastrointestinal tract, resulting in increased frequency of bowel movements and fluid contents. Among these agents, irinotecan (CPT-11) is most commonly associated with CID. Hesperidin (HPD), a flavonoid glycoside found predominantly in citrus fruits, has anti-oxidation properties and anti-inflammation properties that may benefit CID management. Nevertheless, its potential mechanism is still uncertain. In this study, we firstly evaluated the pharmacodynamics of HPD for the treatment of CID in a mouse model, then used network pharmacology and molecular docking methods to excavate the mechanism of HPD in relieving CID, and finally further proved the predicted mechanism through molecular biology experiments. The results demonstrate that HPD significantly alleviated diarrhea, weight loss, colonic pathological damage, oxidative stress, and inflammation in CID mice. In addition, 74 potential targets for HPD intervention in CID were verified by network pharmacology, with the top 10 key targets being AKT1, CASP3, ALB, EGFR, HSP90AA1, MMP9, ESR1, ANXA5, PPARG, and IGF1. The Kyoto Encyclopedia of Genes and Genomes (KEGG) enrichment analysis revealed that the PI3K–Akt pathway, FoxO pathway, MAPK pathway, TNF pathway, and Ras pathway were most relevant to the HPD potential treatment of CID genes. The molecular docking results showed that HPD had good binding to seven apoptosis-related targets, including AKT1, ANXA5, CASP3, HSP90AA1, IGF1, MMP9, and PPARG. Moreover, we verified apoptosis by TdT-mediated dUTP nick-end labeling (TUNEL) staining and immunohistochemistry, and the hypothesis about the proteins above was further verified by Western blotting in vivo experiments. Overall, this study elucidates the potential and underlying mechanisms of HPD in alleviating CID.

## 1. Introduction

According to the International Agency for Research on Cancer’s “Global Cancer Statistics 2018: GLOBOCAN Estimates of Incidence and Mortality Worldwide for 36 Cancers in 185 Countries” in 2018, there were 9.65 million cancer-related deaths and 18.08 million new instances of the disease globally. Asian patients accounted for over 50% of these fatalities and newly diagnosed cases [1]. This significant cancer burden in China and across Asia imposes substantial physical and financial strain on patients and their families. Chemotherapy remains a primary treatment modality for cancer, but it is associated with considerable adverse effects [2].

Chemotherapy-induced diarrhea (CID) is a common and potentially severe side effect, resulting from the toxic effects of chemotherapeutic agents on the gastrointestinal (GI) tract, leading to increased stool frequency and liquidity [3]. CID often occurs during cancer treatment with CPT-11 as the first-line agent [4]. The incidence of CID varies depending on the chemotherapy regimen. For instance, among patients undergoing treatment regimens that include 5-fluorouracil (5-FU) and CPT-11, the incidence of chemotherapy-induced diarrhea (CID) ranges from 30% to 80%. The incidence is particularly high in patients receiving combination therapies or high-dose treatments [5]. CID ranges from mild to severe, characterized by increased stool frequency and liquidity. Severe diarrhea can result in dehydration, electrolyte disturbances, and kidney failure. Patients often experience abdominal cramping, urgency, and in severe cases, fever and bloody stools. The underlying cause of CID is damage to the intestinal mucosa caused by chemotherapeutic agents. This damage disrupts the epithelial barrier, leading to inflammation and altered absorption and secretion in the intestines. Chemotherapy can also modify the gut microbiome, exacerbating the condition [6]. The etiology of CID includes direct toxic effects of chemotherapy on the GI mucosa, resulting in mucositis and subsequent diarrhea. Specific drugs, such as CPT-11, 5-FU, and capecitabine, are known to cause high rates of CID due to their mechanisms of action on rapidly dividing cells in the GI tract [7]. Management of CID involves both preventive and therapeutic approaches. In addition to treatments such as loperamide and phenethylpiperidine, the growth inhibitor analogue octreotide and probiotics, including herbs such as ginger and ginseng, have shown potential benefit in the management of CID [8].

**Figure 1:**
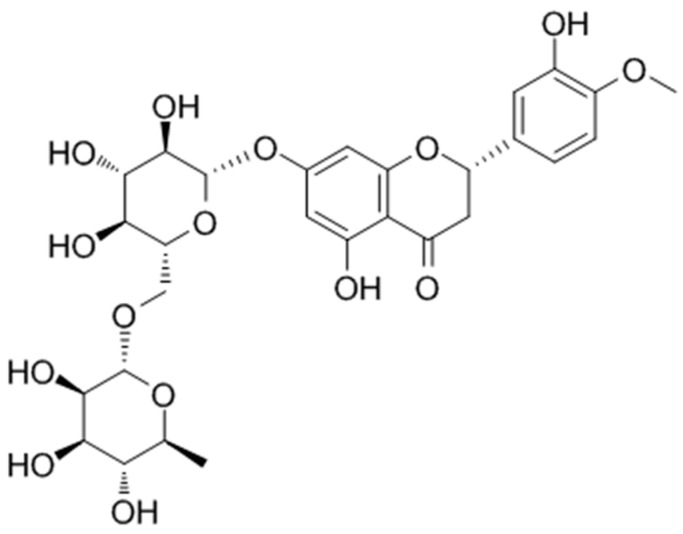
The chemical structure of HPD.

Hesperidin (HPD), a flavonoid glycoside predominantly found in citrus fruits, such as oranges, lemons, and grapefruits [9], has been extensively studied for its health benefits, including antioxidant, anti-inflammatory, and cardiovascular protective effects (Figure 1). It improves vascular function, lowers blood pressure, and supports overall heart health [10]. These effects are primarily attributed to its ability to enhance endothelial function and reduce oxidative stress [11]. HPD also shows promise in treating diarrhea, particularly due to its anti-inflammatory properties. By modulating inflammatory responses in the gut, HPD can reduce the severity and duration of diarrhea [12]. Its ability to improve blood flow and support vascular health also contributes to better gut health, potentially reducing the incidence and severity of diarrhea [13]. However, the mechanism of HPD treatment for CID remains poorly understood and requires further investigation.

Network pharmacology is a multidisciplinary approach that integrates systems biology, bioinformatics, and pharmacology to study the interactions between drugs, targets, and disease networks. This approach enables a more comprehensive understanding of the mechanisms of action of drugs, considering their effects on multiple targets and pathways rather than single-target interactions [14]. In recent years, network pharmacology has gained attention for its potential in drug discovery, particularly in traditional Chinese medicine, where complex herbal formulations involve multiple compounds acting on various targets [15]. For example, studies have demonstrated how network pharmacology can be used to predict potential therapeutic effects, identify bioactive compounds, and explore the mechanisms behind complex diseases like cancer and cardiovascular disorders [16]. By integrating molecular networks, computational tools, and big data, network pharmacology offers a novel approach to precision medicine, moving beyond the “one drug, one target” paradigm [17].

In this study, CPT-11 was utilized to create a CID mouse model to assess the therapeutic efficacy of HPD. In addition, we predicted the key targets and pathways for HPD ameliorating CID by network pharmacology and molecular docking analyses. Further, we confirmed the predicted key proteins and pathways through in vivo experiments. This study informs the discovery of drugs for the treatment of CID and provides novel insights into the therapeutic potential of HPD for CID.

## 2. Results

### 2.1. Amelioration of HPD on CID

#### 2.1.1. HPD Ameliorates Diarrhea in CID Mice

**Figure 2:**
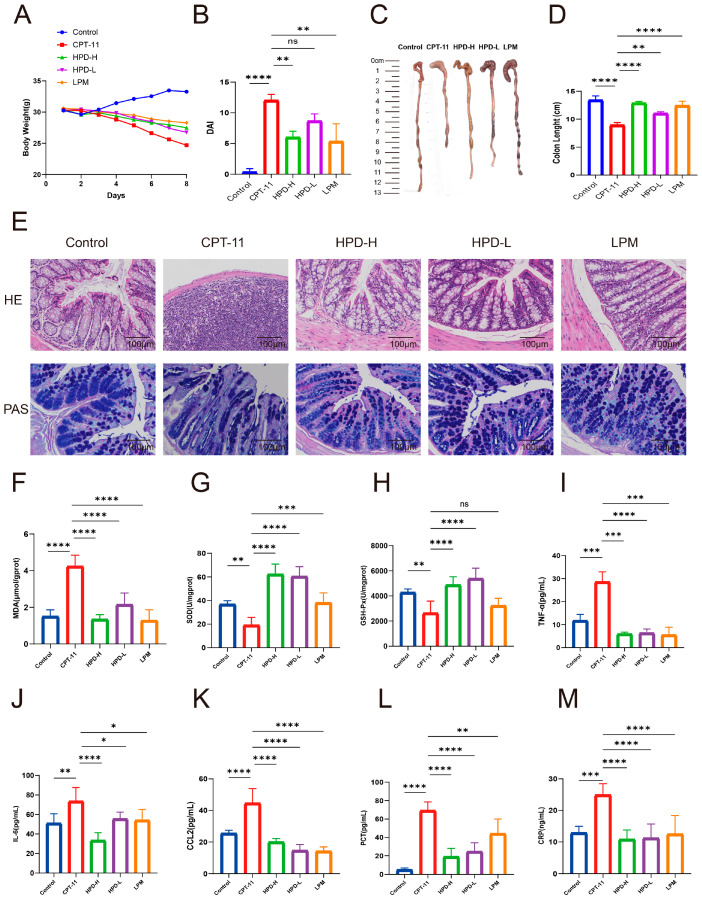
Effects of HPD on mice with CID. (**A**) Body weight; (**B**) disease activity index; (**C**) representative pictures of colon tissues; (**D**) colon length; (**E**) representative H&E staining and PAS staining of colon tissues (magnification, 100×; scale bar, 100 μm); (**F**–**H**) levels of MDA, SOD, and GSH-Px; (**I**–**M**) levels of TNF-α, IL-6, CCL2, PCT, and CRP. Data were presented as the mean ± SD. * *p* < 0.05, ** *p* < 0.01, *** *p* < 0.001, **** *p* < 0.0001 and ns indicates no statistically significant difference (*p* ≥ 0.05).

In this experiment, the control group exhibited normal conditions, whereas the CPT-11 group exhibited poor mental status, lusterless and disheveled fur, reduced food intake, and severe cases of blood in the stool. Moreover, the CPT-11 group displayed decreased body weight, increased DAI scores, and shortened colon length. However, as shown in Figure 2A–D, the variations were significantly improved by HPD treatment.

#### 2.1.2. HPD Attenuates Intestinal Pathology in CID Mice

The H&E staining showed that the CPT-11 group had mucosal epithelial disruption and inflammatory cell infiltration. Nevertheless, HPD obviously transformed the pathological changes (Figure 2E). The PAS staining showed that goblet cells in the control group were full and round, whereas the goblet cells in the CPT-11 group exhibited varying degrees of atrophy and reduction (Figure 2E). The HPD-H, HPD-L, and LMP groups showed reversal of goblet cell atrophy and an increase in mucus secretion.

#### 2.1.3. HPD Attenuates Intestinal Oxidative Stress in CID Mice

Due to the potent antioxidant effect of HPD, we aimed to verify its effects on CID in mice. We monitored the oxidative product MDA and the antioxidant enzymes SOD and GSH-Px (Figure 2F–H). In the CPT-11 group, the MDA levels were elevated; however, this elevation was inhibited by HPD administration. The levels of SOD and GSH-Px decreased in the CPT-11 group, but HPD administration increased the levels of SOD and GSH-Px.

#### 2.1.4. HPD Attenuates Intestinal Inflammation in CID Mice

We also assessed the changes in the levels of inflammatory cytokines. The levels of TNF-α and IL-6 in the CPT-11 group were notably increased, but HPD inhibited this elevation (Figure 2I,J). In addition, the CPT-11 group showed increased expression of CCL2, whereas these levels were markedly lower in the HPD groups (Figure 2K).

To monitor intestinal bacterial infections in the CID mice, PCT and CRP concentrations in the colon tissues were measured. The levels of PCT and CRP were notably higher in the CPT-11 group, but HPD inhibited this elevation (Figure 2L,M). These results demonstrate that HPD could alleviate CID in mice.

### 2.2. The Effect of HPD on CID Analyzed by Network Pharmacology

#### 2.2.1. Network Analysis of Targets

**Figure 3:**
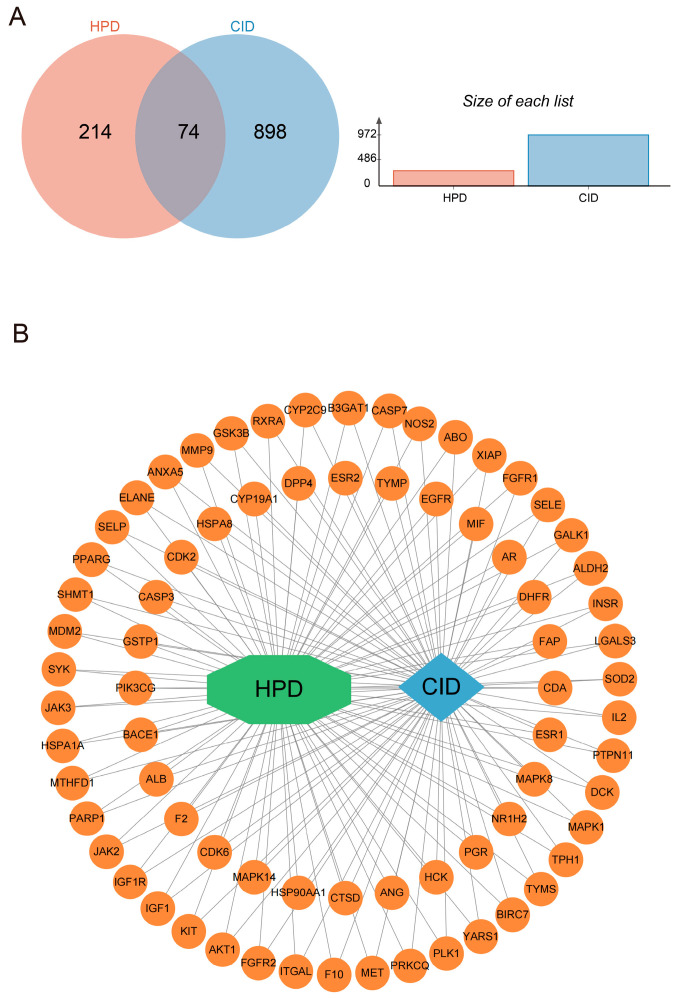
Targets and network analysis of HPD for treating CID. (**A**) Venn diagram of the intersection targets of HPD and CID; (**B**) the regulatory network of HPD acting on CID.

**Figure 4:**
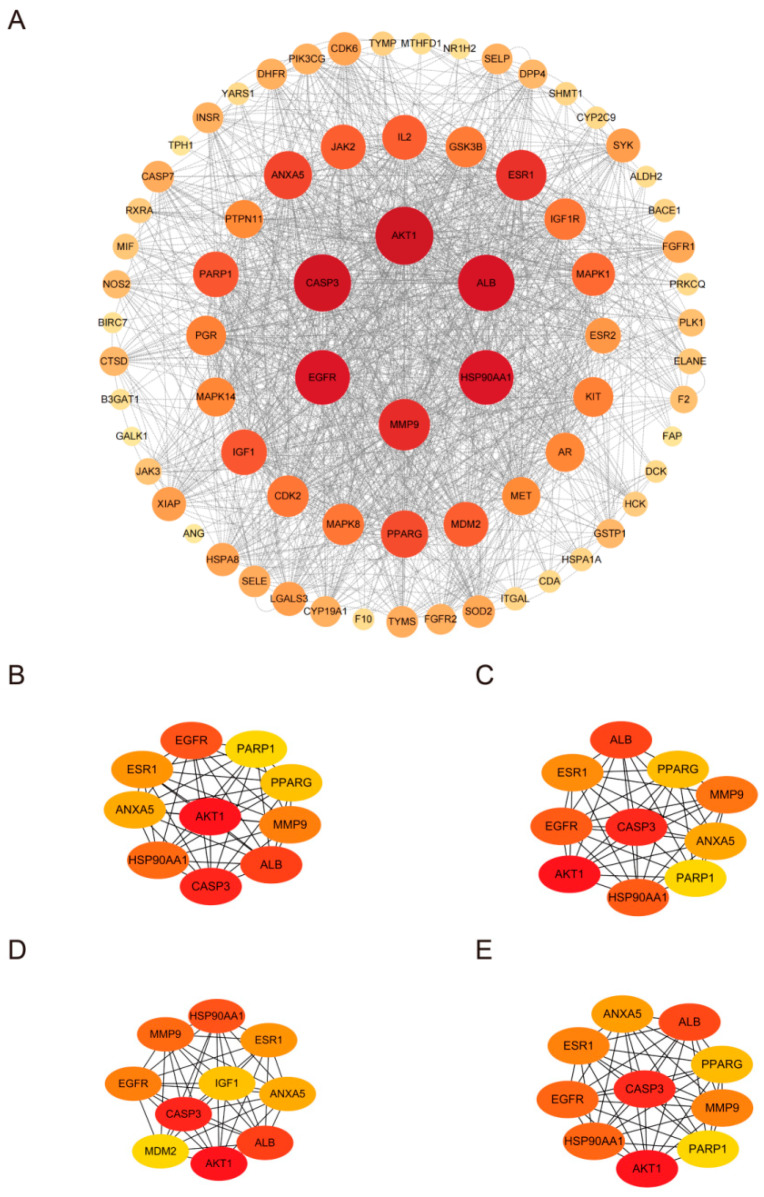
(**A**) PPI network of the potential targets; (**B**) closeness; (**C**) degree; (**D**) MCC; and (**E**) MNC. Deeper colors represent proteins with higher importance in the disease, while lighter colors indicate lower importance.

HPD target genes were identified from the PharmMapper database, excluding genes lacking official gene symbols. Among the 290 active targets identified, 2 duplicates were removed, leaving 288 included in our analysis. A search of the GeneCards and OMIM databases for “chemotherapy-induced diarrhea” yielded 974 active targets. After removing 2 duplicates, 972 valid targets were included in the analysis. A total of 74 overlapping genes between the HPD and CID targets were identified (Figure 3A). The regulatory network of HPD and CID was built by Cytoscape v3.10.1 (San Francisco, CA, USA) (Figure 3B). We built the protein–protein interaction (PPI) network of the proteins mentioned above via STRING. We employed Cytoscape v3.10.1 (San Francisco, CA, USA) for visualization (Figure 4A). This network included 73 nodes and 1318 edges. The top ten core targets were AKT1, CASP3, ALB, EGFR, HSP90AA1, MMP9, ESR1, ANXA5, PPARG, and IGF1. The CytoHubba plugin was subsequently applied to screen for central targets. By integrating the results from the four algorithms, the targets that overlapped across these screening methods were identified as the central targets (Figure 4B–E).

#### 2.2.2. Enrichment Analysis of Overlapped Target

**Figure 5:**
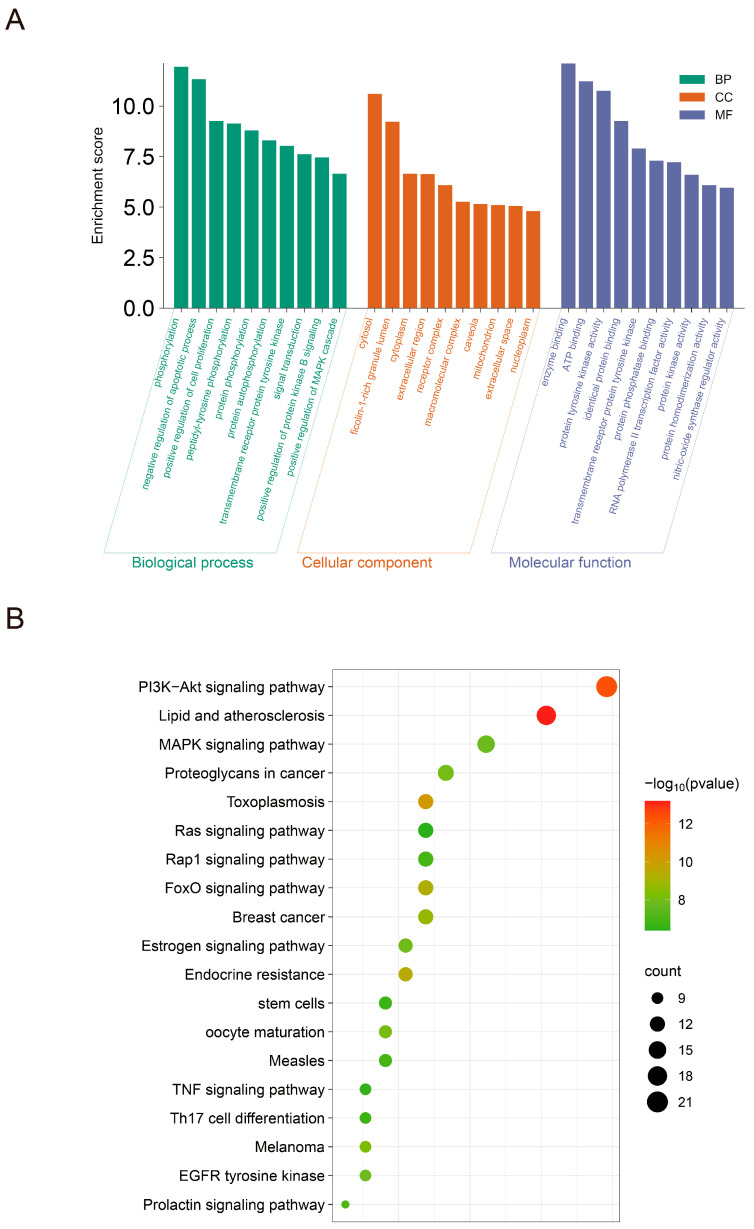
(**A**) Histogram of the GO enrichment analysis involving BP, CC, and MF; (**B**) bubble chart of KEGG pathway.

In the GO enrichment analysis, which included 298 BP terms, 43 CC terms, and 84 MF terms, the top 10 significantly enriched terms were selected for presentation (Figure 5A). The results indicated that HPD’s potential therapeutic genes for CID were predominantly enriched in processes such as phosphorylation, negative regulation of the apoptotic process, and functions including enzyme binding and ATP binding.

The KEGG pathway enrichment analysis identified 135 pathways (*p* < 0.05), from which 20 pathways were found to be significant (*p* < 0.05) (Figure 5B). According to the KEGG analysis, the PI3K–Akt pathway, FoxO pathway, MAPK pathway, TNF pathway, and Ras pathway were most associated with HPD’s potential treatment of CID.

### 2.3. Molecular Docking Analysis

**Figure 6:**
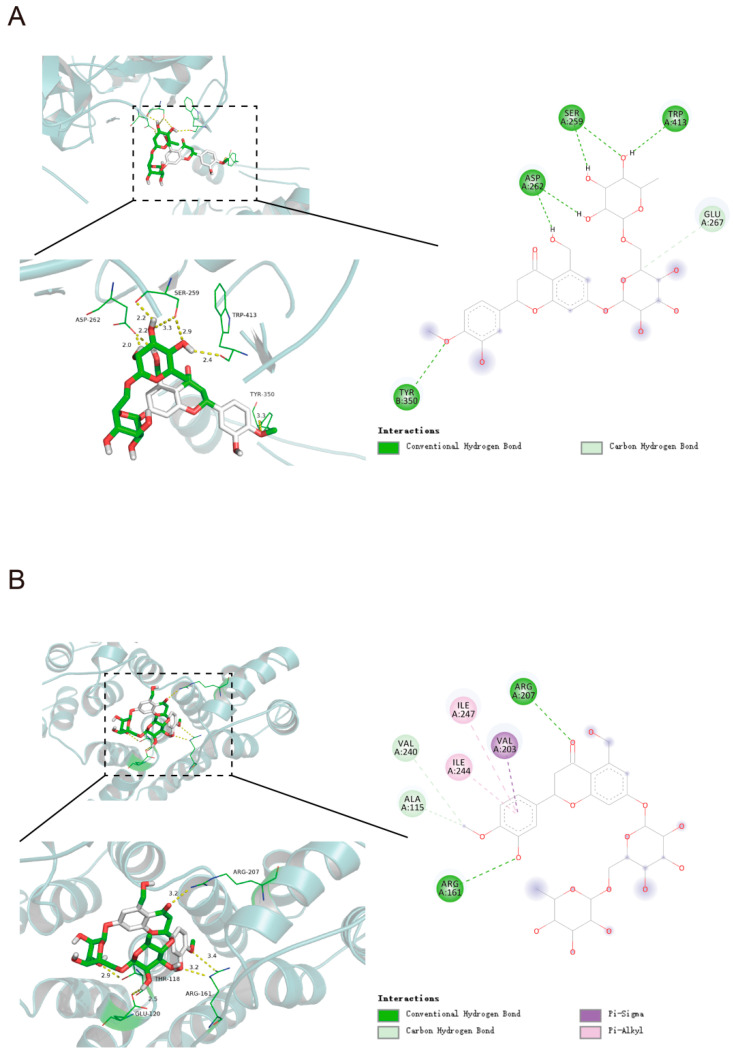

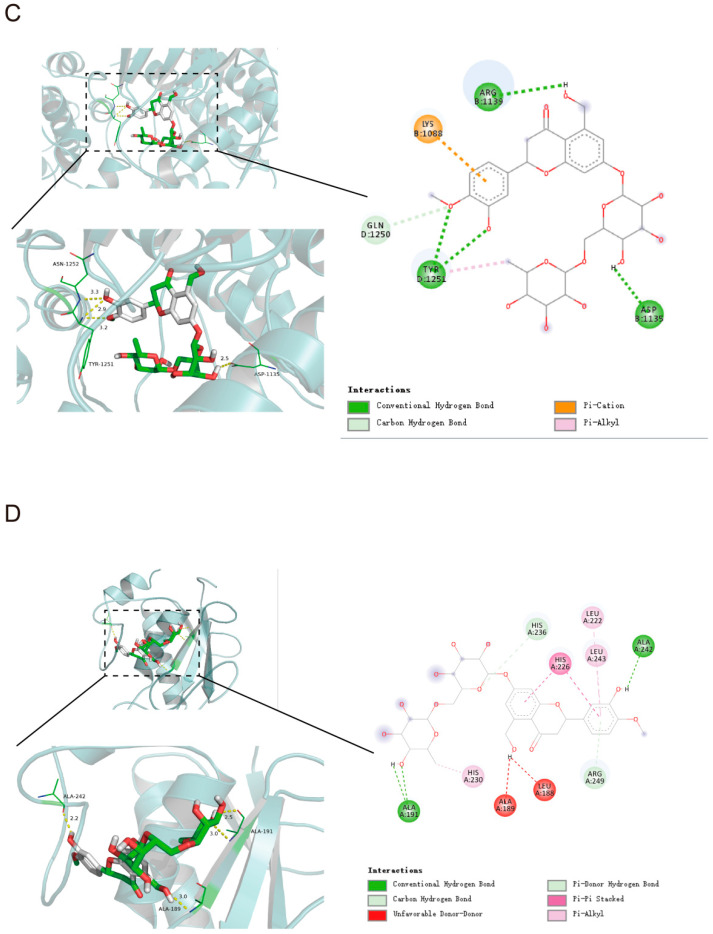

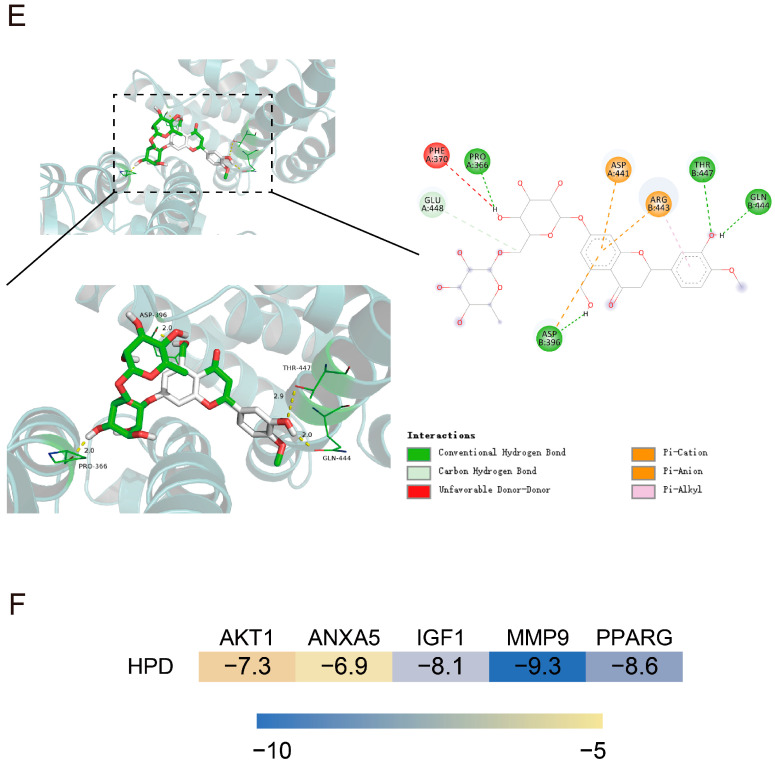
Schematic diagram of the interaction between HPD and target proteins. (**A**) AKT1; (**B**) ANXA5; (**C**) IGF1; (**D**) MMP9; (**E**) PPARG; (**F**) heat map of molecular docking binding energy.

AutoDockTools 1.5.6 software was used for molecular docking, and we explored the interaction and binding modes of HPD with target proteins at the molecular level. HPD was docked into the active pockets of the top ten proteins (AKT1, CASP3, ALB, EGFR, HSP90AA1, MMP9, ESR1, ANXA5, PPARG, and IGF1), and the theoretical binding modes were visualized using PyMOL v1.7.2.1 (New York City, NY, USA). The results demonstrated strong binding of HPD to seven core targets: AKT1, ANXA5, CASP3, HSP90AA1, IGF1, MMP9, and PPARG, with binding free energies of −7.3 kcal/mol, −6.9 kcal/mol, −6.1 kcal/mol, −5.2 kcal/mol, −8.1 kcal/mol, −9.3 kcal/mol, and −8.6 kcal/mol, respectively. The molecules formed a conventional hydrogen bond, carbon hydrogen bond, unfavorable donor–donor, Pi–cation, Pi–anion, Pi–alkyl, Pi–sigma, and Pi–Pi stacked, which highlighted the role of HPD in the regulation of the key targets. We performed a visual analysis of the first five targets (Figure 6). Based on these findings, we predicted that these target proteins may contribute to HPD in relieving CID, prompting further experimental validation.

### 2.4. Verification of HPD on Alleviating CID

#### 2.4.1. HPD Attenuates Enterocyte Apoptosis in CID Mice

**Figure 7:**
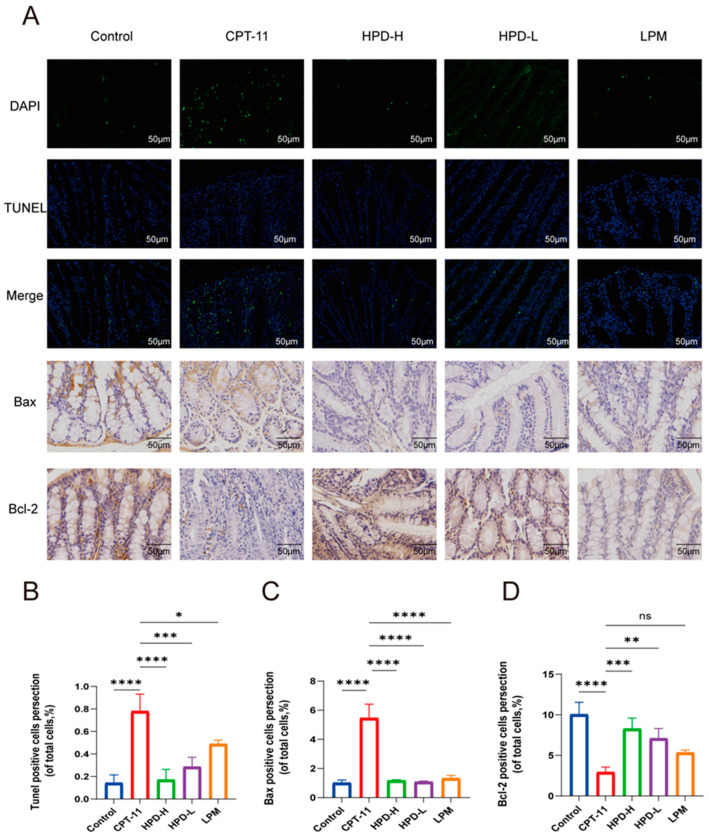
HPD attenuates enterocyte apoptosis in CID mice. (**A**) Representative TUNEL staining of colon tissues, and immunohistochemistry staining of Bax and Bcl-2 protein (magnification, 400×; scale bar, 50 μm). Representative images of the rate of apoptosis by TUNEL staining (**B**), Bax (**C**), and Bcl-2 (**D**) protein level in colon determined by immunohistochemistry. The data were presented as the mean ± SD. * *p* < 0.05, ** *p* < 0.01, *** *p* < 0.001, **** *p* < 0.0001 and ns indicates no statistically significant difference (*p* ≥ 0.05).

The seven proteins above are closely linked to apoptosis, and apoptosis of intestinal epithelial cells is a critical pathological feature leading to damage of the intestinal mucosal barrier [18]. Therefore, we conducted a TUNEL staining assay (Figure 7A,B). The results revealed a significantly higher rate of apoptosis in the CPT-11 group; however, HPD treatment effectively reversed this change.

Furthermore, Bcl-2 and Bax are intricately involved in apoptosis, with Bcl-2 being anti-apoptotic and Bax pro-apoptotic. The reciprocal interactions between these proteins affect cellular survival or death [19]. An immunohistochemistry analysis of Bcl-2 and Bax proteins is presented in Figure 7C,D. In the CPT-11 group, Bax expression was markedly increased, and Bcl-2 expression was decreased, indicating enhanced apoptosis. Treatment with HPD significantly reversed these situations, suggesting its potential to mitigate apoptosis in colonic mucosal cells by modulating Bcl-2 family proteins.

#### 2.4.2. WB Results for 7 Key Proteins

**Figure 8:**
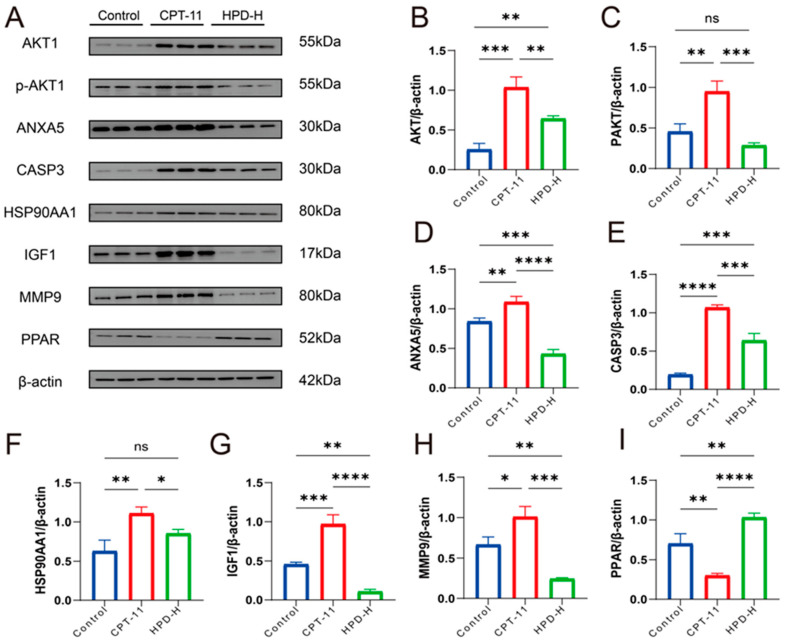
WB results of 7 key proteins. (**A**–**I**) The expression levels of AKT1, PAKT, ANXA5, CASP3, HSP90AA1, IGF1, MMP9, and PPARG in the colon tissue from mice as identified through Western blotting with β-actin as the internal reference. The data were presented as the mean ± SD. * *p* < 0.05, ** *p* < 0.01, *** *p* < 0.001, **** *p* < 0.0001 and ns indicates no statistically significant difference (*p* ≥ 0.05).

According to network pharmacology and molecular docking studies, HPD intervention in CID may be strongly associated with seven core target proteins: AKT1, ANXA5, CASP3, HSP90AA1, IGF1, MMP9, and PPARG. To further validate these findings, we conducted Western blotting experiments (Figure 8). The results demonstrated that in the model group, the expression levels of AKT1, p-AKT1, ANXA5, CASP3, HSP90AA1, and IGF1 were increased, while MMP9 was decreased compared to the control group. Administration of HPD-H markedly reversed these effects in the CPT-11 group, indicating its regulatory effect on these proteins.

## 3. Discussion

CID is a common complication in cancer patients receiving chemotherapy. A study found that 45 ± 2% of 100 patients receiving chemotherapy experienced diarrhea. Among them, 66 adjustments were made during the chemotherapy treatment between 56 patients, including dose reductions in 34%, dose delays in 12%, therapy discontinuations in 23%, or multiple changes in 17% [20]. CID interferes with patients’ daily activities and has a bad impact on patients’ quality of life [21]. Notably, CID often occurs during cancer treatment with CPT-11 as the first-line agent. The mechanism of CPT-11-induced diarrhea is primarily associated with its active metabolite, SN-38. CPT-11 is converted into SN-38, which is highly toxic to the intestinal mucosa, leading to apoptosis and mucosal damage, causing severe diarrhea [22]. The toxic form of SN-38 is stabilized in an acidic environment, increasing its absorption and toxicity in the intestines. Studies suggest that alkalizing the intestinal environment, such as with sodium bicarbonate supplementation, can reduce SN-38 toxicity and alleviate irinotecan-induced diarrhea [23]. HPD comes from a wide range of sources and possesses anti-inflammatory and antioxidant activities, and some studies in the literature have reported that HPD can alleviate CID, so we further investigated the effect and mechanism of HPD in the treatment of CID. The effect of HPD on CID was examined in the study, and we explored the mechanism of HPD intervention in CID using network pharmacology and molecular docking. Additionally, we verified the therapeutic role of HPD on CID. We found that CID causes severe colonic damage, oxidative stress, and inflammatory attacks, all of which were significantly reversed by the administration of HPD.

Oxidative stress is a biological phenomenon resulting from disruptions in the redox state caused by environmental conditions both inside and outside the cell, which lead to excessive production of oxides, such as ROS and MDA, as well as decreased SOD activity, ultimately disrupting oxidative protection mechanisms [24]. GSH-Px is a critical antioxidant that inhibits oxygen free radicals and blocks lipid peroxidation reactions, thereby protecting the body from peroxide-induced damage [25]. Chemotherapy can damage the colonic mucosa, and oxidative stress is pivotal in the pathogenesis and treatment of intestinal damage [26]. It has been reported that intestinal arginine and NO metabolism triggers intestinal barrier dysfunction, low-grade endotoxemia, and aging [27]. Excessive oxidative stress activation in the intestinal mucosa damages the mucosal epithelium and impairs mucosal function, thereby exacerbating the inflammatory response. A study found that cystine (Cys2), a substrate of glutathione (GSH), can locally inhibit oxidative stress-induced pro-inflammatory responses and thus improve intestinal barrier function [28]. Thus, regulating antioxidant defenses is crucial in treating CID. HPD is a potent antioxidant with excellent reducing power and chelating activity. Moreover, its antioxidant activity increases with higher concentrations [29]. The study’s findings demonstrated that HPD intervention significantly reduced MDA levels while increasing SOD and GSH-Px levels. These results indicate that HPD enhances the antioxidant capacity of colon tissues.

CPT-11 is converted to SN-38 by hepatic metabolism, and SN-38 is converted to glucuronidated SN-38 by uridine diphosphate glucuronosyltransferase-1A1 and 1A9 in the liver, and the free SN-38 in the intestinal lumen can induce intestinal mucosal injury in mice [30]. In addition, CID can cause intestinal structural damage to the mucosal barrier by altering the intestinal flora, causing bacterial entry into the bloodstream and inducing inflammation [31]. Among the inflammatory responses induced by CPT-11, production of the pro-inflammatory factors TNF-α and IL-6 causes connective tissue damage and epithelial basal cell death [32]. A study indicated that increased levels of CCL2 in macrophages could promote colitis [33]. HPD exhibits anti-inflammatory effects in both animal models and human trials [34]. Our study shows that the levels of TNF-α, IL-6, and CCL2 were markedly higher in the CPT-11 group, but HPD reversed these aberrant changes. Meanwhile, we measured the concentrations of PCT and CPR in colon tissue to monitor intestinal bacterial infections in CID mice [35]. The results showed that the levels of PCT and CRP were notably higher in the CPT-11 group, but HPD inhibited this elevation.

Based on the effectiveness of HPD for the treatment of CID, we predicted the potential mechanism through network pharmacology. Findings from network pharmacology suggested that the mechanism by which HPD treated CID involved multiple protein targets and pathways: AKT1, CASP3, ALB, EGFR, HSP90AA1, MMP9, ESR1, ANXA5, PPARG, and IGF1 were the top ten core potential targets. GO and KEGG analyses displayed that the relevant pathways were the PI3K–Akt pathway, FoxO pathway, MAPK pathway, TNF pathway, and Ras pathway. Of these, the PI3K–AKT, Ras, and TNF pathways might have influence on HPD intervention in CID via inflammation suppression and anti-carcinogenesis [36]. A study indicated that the molecular signature of the MAPK pathway may be a potential biomarker for evaluating the risk of intestinal damage [37]. Another study found that a FoxO1 inhibitor could significantly reduce intestinal symptoms in an in vivo experiment [38]. To further evaluate the binding ability of HPD to core targets, we performed molecular docking. Seven key targets were screened by molecular docking, including AKT1, ANXA5, CASP3, HSP90AA1, IGF1, MMP9, and PPARG.

Apoptosis of intestinal epithelial cells is an important pathological feature that leads to damage of the intestinal mucosal barrier and triggers diarrhea. It has been reported that CPT-11-induced apoptosis could eliminate peritoneal resident macrophages, which may impair the function of peritoneal B-1 cells to maintain intestinal homeostasis, which is one of the reasons why CPT-11-treated cancer patients can experience diarrhea [39]. Interestingly, the seven proteins above are closely related to apoptosis. Molecular docking screened seven proteins closely related to apoptosis. AKT1 enhances cell proliferation through cell cycle proteins, such as p21, p27, and cyclin D1, and inhibits apoptosis via proteins like p53 [40]. ANXA5 is a cell membrane-associated protein with important roles in apoptosis, coagulation, inflammation, and biomedical fields [41]. MMP9 increases the expression of pro-apoptotic proteins and DNA damage [42]. Caspase-3 [43], HSP90AA1 [44], IGF-1 [45], and PPARG [46] can regulate a variety of apoptotic signaling molecules, such as Bcl-2, to inhibit apoptosis, and these functions are important for maintaining cell survival and removing damaged cells. A recent study showed that HPD modulates NF-κB signaling, PI3/Akt/PTE signaling, and microRNA to improve apoptosis in vivo and in vitro [47]. Meanwhile, our study shows that the protein expression of AKT1, p-AK1, ANXA5, CASP3, HSP90AA1, IGF1, and MMP9 were increased, and PPARG was decreased in the CPT-11 group. However, administration of HPD-H markedly reversed these effects in the CPT-11 group. Therefore, we hypothesize that HPD may alleviate CID by inhibiting the expression of AKT1, ANXA5, CASP3, HSP90AA1, IGF-1, and MMP9, while enhancing the expression of PPARG.

In addition, we observed apoptosis by TUNEL staining and immunohistochemical analysis. The results of the TUNEL staining showed that the rate of apoptosis was notably increased in the CPT-11 group; however, HPD was able to reverse this change. The endogenous apoptosis pathway is mainly executed by the Bcl-2 apoptosis family proteins, of which the pro-apoptotic protein Bax and the anti-apoptotic protein Bcl-2 are the best indicators of apoptosis [48]. The immunohistochemical results showed that the expression of Bax was significantly higher, while Bcl-2 was significantly lower in the CPT-11 group. This result was indicative of apoptosis. HPD treatment reduced the expression of Bax and enhanced the expression of Bcl-2, suggesting that it can attenuate apoptosis in colonic mucosal cells by regulating Bcl-2 family proteins. Despite the prospective findings, this study had several limitations. As a next step, we will further validate the deeper mechanisms through in vitro experiments using inhibitors and agonists on key proteins of the pathway.

## 4. Materials and Methods

### 4.1. Chemicals and Reagents

CPT-11 was supplied by Tianjin JinYao Pharmaceutical Co., Ltd. (Tianjin, China). HPD, with a purity greater than 98%, was sourced from citrus peels and procured from Xi’an Yiyang Bio-TECH (Xi’an, China). The malondialdehyde colorimetric assay kit (MDA, WU148LD87626), total superoxide dismutase activity assay kit (SOD, WU134H066375), glutathione peroxidase activity assay kit (GSH-Px, WU120H4D5123), mouse TNF-α ELISA kit (TNF, WU12P8808590), mouse interleukin 6 ELISA kit (IL-6, WU10042L5917), mouse monocyte chemotactic protein 1 ELISA kit (CCL2, WU11V2R07171), mouse procalcitonin ELISA kit (PCT, WU078V441694), and mouse C-reactive protein ELISA kit (CRP, WU06Z4040286) were from Elabscience Biotechnology Co., Ltd. (Wuhan, China). The AKT1 pAb (AKT, M29N003), IGF1 pAb (IGF1, N25MA23), MMP9 pAb (MMP9, M12DE14), and PPAR gamma pAb (PPAR, M04AP01) were from Chengdu Zen-Bioscience Co., Ltd. (Chengdu, China). The Annexin V pAb (ANXA5, 00107879), Caspase 3/p17/p19 pAb (Caspase-3, 00136098), and HSP90 pAb (HSP90, 00138548) were from Proteintech Group, Inc. (Wuhan, China). The Phospho-Akt mAb (PAKT, 25) was from Cell Signaling Technology, Inc. (Danvers, MA, USA). Other chemicals and reagents were sourced from commercial suppliers.

### 4.2. Experimental Animals

Healthy male ICR mice were obtained from a licensed supplier, SPF (Beijing) Biotechnology Co., Ltd. (Beijing, China). These animals were maintained in plastic cages under controlled environmental conditions: a temperature of 23 ± 2 °C, relative humidity of 50 ± 10%, and a 12 h light/dark cycle. The mice were provided with unrestricted access to food and water. Efforts were made to minimize animal usage and alleviate their distress.

### 4.3. Experimental Design

A total of 60 mice were divided into six groups (*n* = 10 in each group) at random: Control group, CPT-11 group, high-dose HPD (HPD-H) group (80 mg/kg), low-dose HPD (HPD-L) group (20 mg/kg), and loperamide (LPM) group (50 mg/kg). Based on the literature [49], from days 1 to 4, the Control group received intraperitoneal injections of saline, while the other groups received intraperitoneal injections of CPT-11 (40 mg/kg/day). From days 1 to 8, the Control and CPT-11 groups were administered saline orally, while the LPM, HPD-L, and HPD-H groups received the corresponding doses of their respective solutions orally.

### 4.4. Assessment of Severity of CID

The severity of CID was evaluated using the disease activity index (DAI), which is measured by hematochezia, weight loss, and stool consistency. Weight loss is scored from zero to four, with zero points for no change or weight gain, one point for weight loss between 1% and 5%, two points for a loss between 5% and 10%, three points for a loss between 10% and 15%, and four points for a loss exceeding 15%. Stool consistency is rated as zero points for normal stools, two points for loose stools, and four points for diarrhea. Hematochezia is also scored, with zero points indicating no bleeding, two points for positive occult blood, and four points for visible bleeding. The overall DAI value is obtained by summing the scores of these three categories [50].

### 4.5. Histopathological Examination

Colon tissue samples were fixed in 4% paraformaldehyde, embedded in paraffin, and sectioned into 5 μm slices. These sections were then subjected to hematoxylin and eosin (H&E) staining, terminal deoxynucleotidyl transferase dUTP nick-end labeling (TUNEL) staining, and periodic acid-Schiff (PAS) staining for histological analysis. 

### 4.6. ELISA Determination

The colon tissue samples were homogenized in phosphate-buffered saline (PBS) and centrifuged at 3000 rpm for 20 min. The supernatant was subsequently collected, and the optical density (OD) values were measured using an ELISA reader according to the provided instructions. This procedure was employed to quantify the levels of MDA, SOD, GSH-Px, TNF, IL-6, CCL2, PCT, and CRP in the tissue samples.

### 4.7. Network Pharmacology Analysis

#### 4.7.1. HPD Target Prediction and Disease-Related Target Screening

Target prediction for HPD was conducted using the PharmMapper Database [51] (https://www.lilab-ecust.cn/pharmmapper/ Accessed on 5 December 2023), with the species restricted to “*Homo sapiens*”. Additionally, relevant disease targets were identified by inputting the keywords “chemotherapy-induced diarrhea” into the GeneCards Database [52] (http://www.genecards.org/ Accessed on 5 December 2023) and OMIM Database [53] (http://omim.org/ Accessed on 5 December 2023). Subsequently, false-positive and duplicate targets were removed and integrated. The intersection of HPD targets and CID targets was determined using R v4.3.2 (Vienna, Austria) packages to identify potential HPD targets for treating the disease. The common gene targets were visualized in a Venn diagram [54] (https://www.bioinformatics.com.cn/ Accessed on 5 December 2023). Finally, Cytoscape v3.10.1 (San Francisco, CA, USA) [55] was utilized to construct the regulatory network of HPD’s action on CID.

#### 4.7.2. Protein–Protein Interaction Network Analysis

The protein–protein interaction (PPI) network of the target genes was retrieved from the Search Tool for the Retrieval of Interacting Genes/Proteins (STRING) database [56] (https://cn.string-db.org/ Accessed on 5 December 2023.) with a minimum required interaction score of ≥0.4, restricted to the species “*Homo sapiens*”. The network visualization was conducted using Cytoscape v3.10.1 (San Francisco, CA, USA). In this PPI network, nodes denote the target proteins, and edges represent the predicted or experimentally validated interactions between these proteins. To identify core targets related to CID, the CytoHubba plugin in Cytoscape v3.10.1 (San Francisco, CA, USA) was employed using the closeness, degree, maximal clique centrality (MCC), and maximum neighborhood component (MNC) algorithms. The top 10 targets identified by each method were analyzed, and the overlapping targets among these methods were considered the central targets [57].

#### 4.7.3. GO and KEGG Pathway Enrichment Analyses

Gene ontology (GO) enrichment analysis encompasses biological process (BP), molecular function (MF), and cellular component (CC) categories. The Kyoto Encyclopedia of Genes and Genomes (KEGG) serves as a bioinformatics resource for identifying significantly altered metabolic pathways enriched in the gene list. The clusterProfiler package in R v4.3.2 (Vienna, Austria) was utilized to conduct a GO enrichment analysis (*p* < 0.05) and a KEGG pathway analysis (*p* < 0.05) on the target genes of HPD acting on CID. The results were visualized using the bioinformatics platform [58] (http://www.bioinformatics.com.cn/). 

### 4.8. Molecular Docking

Based on the results from the previous network pharmacology screening, the 3D structures of the top ten target proteins were retrieved from the protein database (PDB) [59] (http://www.rcsb.org/ Accessed on 10 December 2023) and the corresponding PDB format files were downloaded. PyMOL v1.7.2.1 (New York City, NY, USA) [59] was employed to remove ligands, ions, and solvent molecules from these structures. The chemical structure of HPD (PubChem CID 10621) was obtained from the PubChem database [60] (https://pubchem.ncbi.nlm.nih.gov/ Accessed on 10 December 2023). Additionally, AutoDockTools 1.5.6 software was used to convert the formats of HPD and the target genes to pdbqt format for molecular docking. In molecular docking, the selection of protein crystals and binding sites is critical for ensuring docking accuracy. High-resolution structures with a resolution below 2.5 Å should be prioritized for structural precision [61]. It is essential to consider the integrity of the protein, prioritizing full-length proteins or those containing key functional domains while avoiding excessive mutations or modifications. Additionally, structures bound with known ligands help capture active conformations [23]. For selecting binding sites, known active sites should be prioritized; in their absence, homology modeling, pocket detection tools, and molecular dynamics simulations can be used to predict and validate potential binding pockets [62].

### 4.9. Immunohistochemical Analysis

The colon paraffin sections were routinely deparaffinized and washed three times with distilled water for 3 min each time; then, the endogenous peroxidase was sealed with 3% hydrogen peroxide, the sections were incubated with microwave heating antigenic repair of 10% normal goat serum, drops of p53 antibody diluted 1:100 with PBS were added, and the samples were stored overnight in a refrigerator at 4 °C. Goat anti-rabbit lgG secondary antibody was added dropwise by oscillation and washed with PBS, the diamine benzylamine was developed for color development, the hematoxylin was restained, and the sections were routinely mounted. The film was blocked routinely. After observation, photos were taken, and the images were analyzed by Image J v1.54f (Bethesda, MD, USA).

### 4.10. Western Blotting

The colon tissue was homogenized in lysis buffer containing protease inhibitors thoroughly, then centrifuged at 4 °C (12,000 rpm, 15 min). We collected the supernatant and measured the protein concentration using a BCA protein assay kit. The samples were incubated in a metal bath at 95 °C for 15 min. We then separated the protein samples by electrophoresis and transferred them to a PVDF membrane. After blocking for 1 h, we then applied the primary antibodies: Beta-actin (1:10,000), GAPDH (1:10,000), AKT1 (1:3000), ANXA5 (1:3000), Caspase-3 (1:1000), HSP90 (1:3000), IGF1 (1:1000), MMP9 (1:1000), PPAR (1:1000), and PAKT (1:1000). The samples were incubated at 4 °C overnight. We incubated the PVDF membrane with the corresponding secondary antibodies for 1 h, then developed and exposed them using the ChemiScope 6100 chemiluminescence imaging analysis system. We then analyzed the grayscale using Image J v1.54f (Bethesda, MD, USA).

### 4.11. Data Analysis

All the data were expressed as mean ± SD. Comparisons among multiple groups were conducted using one-way analysis of variance (ANOVA). A *p*-value of <0.05 was considered statistically significant. Statistical analyses and graphing were performed using GraphPad Prism 9.0 software (San Diego, CA, USA).

## 5. Conclusions

During the study, we found that HPD notably alleviated CID in mice. Then, a network pharmacology analysis displayed that HPD modulates five pathways associated with CID. Furthermore, a molecular docking analysis indicated that HPD interacts with seven key targets (AKT1, ANXA5, CASP3, HSP90AA1, IGF1, MMP9, PPARG) involved in the regulation of apoptosis. It was supported that HPD ameliorated apoptosis and modulated these seven critical targets by in vivo experiments. In summary, these findings provide preliminary scientific evidence supporting the use of HPD in treating CID. Moreover, we plan to follow up with further research to explore the mechanism of HPD for CPT-11-induced diarrhea.

## Data Availability

Raw data relevant to the conclusions of this study will be provided by the corresponding authors upon reasonable request.

## Abbreviations

5-FU, 5-fluorouracil; BP, biological process; CC, cellular component; CCL2, C-C motif chemokine ligand 2; CID, chemotherapy-induced diarrhea; CRP, C-reactive; HPD, hesperidin; DAI, disease activity index; GO, gene ontology; GSH-Px, glutathione peroxidase; H&E, hematoxylin and eosin; IL-6, interleukin-6; KEGG, Kyoto Encyclopedia of Genes and Genomes; LPM, loperamide; MDA, malondialdehyde; MF, molecular functions; NO, nitric oxide; OD, optical density; PAS, periodic acid-Schiff; PBS, phosphate-buffered saline; PCT, procalcitonin; PPI, protein–protein interaction; ROS, reactive oxygen species; SD, standard deviation; SOD, superoxide dismutase; TNF-α, tumor necrosis factor-α; TUNEL, TdT-mediated dUTP nick-end labeling; GI, gastrointestinal.

## References

[1] Bray F., Ferlay J., Soerjomataram I., Siegel R.L., Torre L.A., Jemal A. (2018). Global cancer statistics 2018: GLOBOCAN estimates of incidence and mortality worldwide for 36 cancers in 185 countries. CA Cancer J. Clin..

[2] Zraik I.M., Heß-Busch Y. (2021). [Management of chemotherapy side effects and their long-term sequelae]. Urologe.

[3] Maroun J.A., Anthony L.B., Blais N., Burkes R., Dowden S.D., Dranitsaris G., Samson B., Shah A., Thirlwell M.P., Vincent M.D. (2007). Prevention and management of chemotherapy-induced diarrhea in patients with colorectal cancer: A consensus statement by the Canadian Working Group on Chemotherapy-Induced Diarrhea. Curr. Oncol..

[4] Lv J., Jia Y., Li J., Kuai W., Li Y., Guo F., Xu X., Zhao Z., Lv J., Li Z. (2019). Gegen Qinlian decoction enhances the effect of PD-1 blockade in colorectal cancer with microsatellite stability by remodelling the gut microbiota and the tumour microenvironment. Cell Death Dis..

[5] Jansman F.G., Sleijfer D.T., de Graaf J.C., Coenen J.L., Brouwers J.R. (2001). Management of chemotherapy-induced adverse effects in the treatment of colorectal cancer. Drug Saf..

[6] Kawasaki Y., Kakimoto K., Tanaka Y., Shimizu H., Nishida K., Numa K., Kinoshita N., Tatsumi Y., Nakazawa K., Koshiba R. (2023). Relationship between Chemotherapy-Induced Diarrhea and Intestinal Microbiome Composition. Digestion.

[7] Wadler S., Benson A.B., Engelking C., Catalano R., Field M., Kornblau S.M., Mitchell E., Rubin J., Trotta P., Vokes E. (1998). Recommended guidelines for the treatment of chemotherapy-induced diarrhea. J. Clin. Oncol..

[8] Yu Y., Kong R., Cao H., Yin Z., Liu J., Nan X., Phan A.T., Ding T., Zhao H., Wong S.T.C. (2018). Two birds, one stone: Hesperetin alleviates chemotherapy-induced diarrhea and potentiates tumor inhibition. Oncotarget.

[9] Pyrzynska K.A.-O. (2022). Hesperidin: A Review on Extraction Methods, Stability and Biological Activities. Nutrients.

[10] Abuelsaad A.S., Mohamed I., Allam G., Al-Solumani A.A. (2013). Antimicrobial and immunomodulating activities of hesperidin and ellagic acid against diarrheic Aeromonas hydrophila in a murine model. Life Sci..

[11] Habauzit V., Morand C. (2012). Evidence for a protective effect of polyphenols-containing foods on cardiovascular health: An update for clinicians. Ther. Adv. Chronic Dis..

[12] Xu L., Yang Z.L., Li P., Zhou Y.Q. (2009). Modulating effect of Hesperidin on experimental murine colitis induced by dextran sulfate sodium. Phytomedicine.

[13] Shafik N.A.-O., Gaber R.A., Mohamed D.A., Ebeid A.M. (2019). Hesperidin modulates dextran sulfate sodium-induced ulcerative colitis in rats: Targeting sphingosine kinase-1- sphingosine 1 phosphate signaling pathway, mitochondrial biogenesis, inflammation, and apoptosis. J. Biochem. Mol. Toxicol..

[14] Hopkins A.L. (2008). Network pharmacology: The next paradigm in drug discovery. Nat. Chem. Biol..

[15] Zhang X., Zhang J., Zhou Z., Xiong P., Cheng L., Ma J., Wen Y., Shen T., He X., Wang L. (2024). Integrated network pharmacology, metabolomics, and transcriptomics of Huanglian-Hongqu herb pair in non-alcoholic fatty liver disease. J. Ethnopharmacol..

[16] Zhou Z., Chen B., Chen S., Lin M., Chen Y., Jin S., Chen W., Zhang Y. (2020). Applications of Network Pharmacology in Traditional Chinese Medicine Research. Evid. Based Complement. Altern. Med..

[17] Zhao L., Zhang H., Li N., Chen J., Xu H., Wang Y., Liang Q. (2023). Network pharmacology, a promising approach to reveal the pharmacology mechanism of Chinese medicine formula. J. Ethnopharmacol..

[18] Wang Y., Wang Y., Shen W., Wang Y., Cao Y., Nuerbulati N., Chen W., Lu G., Xiao W., Qi R. (2020). Grape Seed Polyphenols Ameliorated Dextran Sulfate Sodium-Induced Colitis via Suppression of Inflammation and Apoptosis. Pharmacology.

[19] Hu Q., Zhang W., Wu Z., Tian X., Xiang J., Li L., Li Z., Peng X., Wei S., Ma X. (2021). Baicalin and the liver-gut system: Pharmacological bases explaining its therapeutic effects. Pharmacol. Res..

[20] Arbuckle R.B., Huber S.L., Zacker C. (2000). The consequences of diarrhea occurring during chemotherapy for colorectal cancer: A retrospective study. Oncologist.

[21] Richardson G., Dobish R. (2007). Chemotherapy induced diarrhea. J. Oncol. Pharm. Pract..

[22] Ikegami T., Ha L., Arimori K., Latham P., Kobayashi K., Ceryak S., Matsuzaki Y., Bouscarel B. (2002). Intestinal alkalization as a possible preventive mechanism in irinotecan (CPT-11)-induced diarrhea. Cancer Res..

[23] Huang S.Y., Zou X. (2010). Advances and challenges in protein-ligand docking. Int. J. Mol. Sci..

[24] Cheng Y., Zan J., Song Y., Yang G., Shang H., Zhao W. (2018). Evaluation of intestinal injury, inflammatory response and oxidative stress following intracerebral hemorrhage in mice. Int. J. Mol. Med..

[25] Izadparast F., Riahi-Zajani B., Yarmohammadi F., Hayes A.W., Karimi G. (2022). Protective effect of berberine against LPS-induced injury in the intestine: A review. Cell Cycle..

[26] Zhang M., Xia F., Xia S., Zhou W., Zhang Y., Han X., Zhao K., Feng L., Dong R., Tian D. (2022). NSAID-Associated Small Intestinal Injury: An Overview from Animal Model Development to Pathogenesis, Treatment, and Prevention. Front. Pharmacol..

[27] Brandt A., Baumann A., Hernández-Arriaga A., Jung F., Nier A., Staltner R., Rajcic D., Schmeer C., Witte O.W., Wessner B. (2022). Impairments of intestinal arginine and NO metabolisms trigger aging-associated intestinal barrier dysfunction and ‘inflammaging’. Redox Biol..

[28] Hasegawa T., Mizugaki A., Inoue Y., Kato H.A.-O.X., Murakami H. (2021). Cystine reduces tight junction permeability and intestinal inflammation induced by oxidative stress in Caco-2 cells. Amino Acids.

[29] Ahmadi A., Shadboorestan A. (2016). Oxidative stress and cancer; the role of hesperidin, a citrus natural bioflavonoid, as a cancer chemoprotective agent. Nutr. Cancer.

[30] Bailly C. (2019). Irinotecan: 25 years of cancer treatment. Pharmacol. Res..

[31] Di Tommaso N., Gasbarrini A., Ponziani F.A.-O. (2021). Intestinal Barrier in Human Health and Disease. Int. J. Environ. Res. Public Health.

[32] Chen J., Li M., Chen R., Xu Z., Yang X., Gu H., Zhang L., Fu C., Zhang J., Wu Y. (2023). Gegen Qinlian standard decoction alleviated irinotecan-induced diarrhea via PI3K/AKT/NF-κB axis by network pharmacology prediction and experimental validation combination. Chin. Med..

[33] He J., Song Y., Li G., Xiao P., Liu Y., Xue Y., Cao Q., Tu X., Pan T., Jiang Z. (2019). Fbxw7 increases CCL2/7 in CX3CR1hi macrophages to promote intestinal inflammation. J. Clin. Invest..

[34] Miles E.A., Calder P.C. (2021). Effects of Citrus Fruit Juices and Their Bioactive Components on Inflammation and Immunity: A Narrative Review. Front. Immunol..

[35] Pierrakos C., Velissaris D., Bisdorff M., Marshall J.C., Vincent J.A.-O. (2020). Biomarkers of sepsis: Time for a reappraisal. Crit. Care.

[36] Liu X., Fan Y., Du L., Mei Z., Fu Y. (2021). In Silico and In Vivo Studies on the Mechanisms of Chinese Medicine Formula (Gegen Qinlian Decoction) in the Treatment of Ulcerative Colitis. Front. Pharmacol..

[37] Zhao X., Kang B., Lu C., Liu S., Wang H., Yang X., Chen Y., Jiang B., Zhang J., Lu Y. (2011). Evaluation of p38 MAPK pathway as a molecular signature in ulcerative colitis. J. Proteome Res..

[38] Wei Y., Fan Y., Huang S., Lv J., Zhang Y., Hao Z. (2024). Baizhu shaoyao decoction restores the intestinal barrier and brain-gut axis balance to alleviate diarrhea-predominant irritable bowel syndrome via FoxO1/FoxO3a. Phytomedicine.

[39] Huang M.Y., Pan H., Liang Y.D., Wei H.X., Xu L.H., Zha Q.B., He X.H., Ouyang D.Y. (2016). Chemotherapeutic agent CPT-11 eliminates peritoneal resident macrophages by inducing apoptosis. Apoptosis.

[40] Hinz N., Jücker M.A.-O. (2019). Distinct functions of AKT isoforms in breast cancer: A comprehensive review. Cell Commun. Signal.

[41] Li Y.Z., Wang Y.Y., Huang L., Zhao Y.Y., Chen L.H., Zhang C. (2022). Annexin A protein family in atherosclerosis. Clin. Chim. Acta.

[42] Lei K., Wei Q., Cheng Y., Wang Z., Wu H., Zhao F., Ding W., Shi F. (2023). OONO-/MMP2/MMP9 pathway-mediated apoptosis of porcine granulosa cells is associated with DNA damage. Reproduction.

[43] Porter A.G., Jänicke R.U. (1999). Emerging roles of caspase-3 in apoptosis. Cell Death Differ..

[44] Yan H., Li Y., Yang B., Long F., Yang Z., Tang D. (2023). Exploring the mechanism of action of Yiyi Fuzi Baijiang powder in colorectal cancer based on network pharmacology and molecular docking studies. Biotechnol. Genet. Eng. Rev..

[45] Gobé G., Zhang X.J., Cuttle L., Pat B., Willgoss D., Hancock J., Barnard R., Endre R.B. (1999). Bcl-2 genes and growth factors in the pathology of ischaemic acute renal failure. Immunol. Cell Biol..

[46] Yang J. (2013). PPAR-γ silencing inhibits the apoptosis of A549 cells by upregulating Bcl-2. Chin. J. Lung Cancer.

[47] Muhammad T.A.-O., Ikram M., Ullah R., Rehman S.U., Kim M.O. (2019). Hesperetin, a Citrus Flavonoid, Attenuates LPS-Induced Neuroinflammation, Apoptosis and Memory Impairments by Modulating TLR4/NF-κB Signaling. Nutrients.

[48] Ashkenazi A., Fairbrother W.J., Leverson J.D., Souers A.J. (2017). From basic apoptosis discoveries to advanced selective BCL-2 family inhibitors. Nat. Rev. Drug Discov..

[49] Wang J., Feng W., Zhang S., Chen L., Tang F., Sheng Y., Ao H., Peng C. (2019). Gut microbial modulation in the treatment of chemotherapy-induced diarrhea with Shenzhu Capsule. BMC Complement. Altern. Med..

[50] Li W., Zhang Y., Wang Q., Wang Y., Fan Y., Shang E., Jiang S., Duan J. (2024). 6-Gingerol ameliorates ulcerative colitis by inhibiting ferroptosis based on the integrative analysis of plasma metabolomics and network pharmacology. Food Funct..

[51] Wang X., Shen Y., Wang S., Li S., Zhang W., Liu X., Lai L., Pei J., Li H. (2017). *PharmMapper* 2017 update: A web server for potential drug target identification with a comprehensive target pharmacophore database. Nucleic Acids Res..

[52] Rebhan M., Chalifa-Caspi V., Prilusky J., Lancet D. (1997). GeneCards: Integrating information about genes, proteins and diseases. Trends Genet..

[53] Amberger J.S., Bocchini C.A., Scott A.F., Hamosh A. (2019). OMIM.org: Leveraging knowledge across phenotype-gene relationships. Nucleic Acids Res..

[54] Bardou P., Mariette J., Escudié F., Djemiel C., Klopp C. (2014). jvenn: An interactive Venn diagram viewer. BMC Bioinform..

[55] Shannon P., Markiel A., Ozier O., Baliga N.S., Wang J.T., Ramage D., Amin N., Schwikowski B., Ideker T. (2003). Cytoscape: A software environment for integrated models of biomolecular interaction networks. Genome Res..

[56] Szklarczyk D., Gable A.L., Lyon D., Junge A., Wyder S., Huerta-Cepas J., Simonovic M., Doncheva N.T., Morris J.H., Bork P. (2019). STRING v11: Protein-protein association networks with increased coverage, supporting functional discovery in genome-wide experimental datasets. Nucleic Acids Res..

[57] Cao Y., Wang C., Dong L. (2023). Exploring the Mechanism of White Peony in the Treatment of Lupus Nephritis Based on Network Pharmacology and Molecular Docking. Arch. Esp. Urol..

[58] Tang D., Chen M., Huang X., Zhang G., Zeng L., Zhang G., Wu S., Wang Y. (2023). SRplot: A free online platform for data visualization and graphing. PLoS ONE.

[59] Burley S.K., Bhikadiya C., Bi C., Bittrich S., Chen L., Crichlow G.V., Christie C.H., Dalenberg K., Di Costanzo L., Duarte J.M. (2021). RCSB Protein Data Bank: Powerful new tools for exploring 3D structures of biological macromolecules for basic and applied research and education in fundamental biology, biomedicine, biotechnology, bioengineering and energy sciences. Nucleic Acids Res..

[60] Seeliger D., de Groot B.L. (2010). Ligand docking and binding site analysis with PyMOL and Autodock/Vina. J. Comput. Aided Mol. Des..

[61] Wang R., Fang X., Lu Y., Wang S. (2017). The PDBbind database: Collection of binding affinities for protein-ligand complexes with known three-dimensional structures. J. Med. Chem..

[62] Kozakov D., Grove L.E., Hall D.R., Bohnuud T., Mottarella S.E., Luo L., Xia B., Beglov D., Vajda S. (2015). The FTMap family of web servers for determining and characterizing ligand-binding hot spots of proteins. Nat. Protoc..

